# The Association between the Substitution of Red Meat with Legumes and the Risk of Primary Liver Cancer in the UK Biobank: A Cohort Study

**DOI:** 10.3390/nu16152383

**Published:** 2024-07-23

**Authors:** Niels Bock, Fie Langmann, Luke W. Johnston, Daniel B. Ibsen, Christina C. Dahm

**Affiliations:** 1 Department of Public Health, Aarhus University, 8000 Aarhus, Denmark; nb@ph.au.dk (N.B.); fie@ph.au.dk (F.L.); lwjohnst@clin.au.dk (L.W.J.); dbi@ph.au.dk (D.B.I.); 2 Steno Diabetes Center Aarhus, Aarhus University Hospital, 8200 Aarhus, Denmark

**Keywords:** food substitutions, liver cancer, red meat, legumes

## Abstract

Primary liver cancer is globally on the rise, partially due to poor diets and sedentary lifestyles. Shifting to more plant-based diets may lower the risk. We aimed to estimate the effect of replacing total red meat, unprocessed red meat and processed red meat with legumes on primary liver cancer in a free-living population. We analyzed data from 126,744 UK Biobank participants who completed ≥ two 24 h diet recalls. Baseline characteristics were collected from the initial assessment visit. Information on liver cancer diagnoses was collected via external linkage to inpatient hospital episodes or central cancer registries. Cox proportional hazards regression models were used to estimate the substitution of 15 g/day of legumes with 15 g/day of total red meat, unprocessed red meat or processed red meat on liver cancer risk, using the leave-one-out food substitution model. During a median follow-up time of 11.1 years, 173 participants developed liver cancer. In the fully adjusted models, no association was observed when substituting 15 g/day of legumes with total red meat (HR: 1.02 (95% CI 0.96–1.08)), unprocessed red meat (HR: 1.00 (95% CI 0.94–1.06)) or processed red meat (HR: 1.09 (95% CI 0.99–1.21)). Overall, little evidence of an association between replacing red meat with legumes and liver cancer was observed. Further research in other study populations with longer follow-up time is warranted.

## 1. Introduction

Hepatocellular carcinoma (HCC) is the sixth most common cancer in the world and the third leading cause of cancer-related death, with viral hepatitis being the leading risk factor [1]. In low-infection populations, modifiable risk factors, such as dietary habits, may play an increasing role in HCC pathogenesis, as non-alcoholic fatty liver disease (NAFLD) has become the leading cause of liver cirrhosis [2,3] which may in turn progress to HCC. A western dietary pattern high in fats and red meats and concurrently low in fruits, vegetables and whole grains has been associated with NAFLD progression [4]. The prevalence of NAFLD-related HCC cases is an increasing global problem [2]. It is estimated that the prevalence of NAFLD-related HCC in the US will increase by 146%, while incident NAFLD-related HCC cases will increase by 137% by 2030 [5].

The second most common primary liver cancer is intrahepatic cholangiocarcinoma (ICC) [6]. While HCC emerges from the liver parenchyma, ICC emerges from the bile duct. Despite being a relatively rare cancer, ICC is characterized by its aggressiveness, late diagnosis and poor survival [7]. It is estimated that the incidence of ICC is increasing in populations that are not burdened by known infectious and environmental risk factors [8]. Recent meta-analyses of observational studies and clinical trials have shown a significant adverse association between NAFLD and ICC [9,10].

The impact of specific food groups on liver cancer risk is not well known. Observational studies suggest that the intake of coffee, vegetables and whole grains may lower HCC risk [11,12,13,14]. The protective properties of these foods are proposed to be their contents of dietary fibers and polyphenols, which are also defining components of legumes [15]. Polyphenols such as phenolic acid and flavonoids inhibit free radicals and may thus protect human tissues against oxidative stress [16,17]. Phytic acid and saponins may have anticarcinogenic properties via regulation of the cell cycle and enzymes involved in the apoptosis pathway and inhibition of the metastatic potential of tumors [15,18,19]. The health benefits of legumes extend to improved glycemic control and hypotensive and anticarcinogenic properties, with observed inverse associations with cardiovascular disease and colorectal cancer [20,21]. Two large prospective cohort studies (N = 130,000–500,000, n event = 298–940) found evidence of inverse associations between legume consumption and risk of HCC [11,13]. However, replacement foods were not specified in these studies, which fails to reflect that a higher intake of one food is at the expense of a concomitantly lower intake of another food. On the contrary, processed red meat intake, but not unprocessed red meat intake, was associated with an increased risk of HCC in two large cohorts (N = 50,000–120,000, n events = 163), suggesting that the processing of red meat may augment the carcinogenic effects on the liver tissues [22]. Processed red meat is classified as “carcinogenic to humans” and unprocessed red meat as a possible carcinogen by the International Agency for Research on Cancer [23]. Studies on substituting animal-based proteins with plant-based proteins are important if we are to lower the climate impacts of our diets [24]. While previous research has investigated and found protective effects when substituting animal-based proteins with plant-based proteins in relation to NAFLD [25], research on substituting meats with legumes in relation to the risk of HCC and ICC is sparse. This leaves a substantial gap in the current knowledge on the beneficial effects on primary liver cancer from substituting red meat with legumes.

The low incidence of liver cancer in populations not burdened by viral hepatitis complicates observational prospective research designs; nonetheless, the prospects of the burden of liver cancer on public health warrant investigation of preventative measures. Thus, the main aim of this study was to estimate the association between replacing unprocessed red meat, processed red meat and total red meat with legumes and primary liver cancer in a large free-living population.

## 2. Materials and Methods

The protocol for this study was written prior to conducting the analysis. It is available on the archive Zenodo [26]. Some changes were made to the final analysis plan due to the lack of power to conduct subgroup and mediation analyses.

### 2.1. Study Population

The UK Biobank is a population-based prospective cohort initiated in 2006 [27]. During 2006–2010, more than 500,000 participants, aged 40–69, were recruited and visited designated assessment centers across the UK. Participants provided information about age, sex, sociodemographic factors and lifestyle factors via touch screen questionnaires and computer-assisted interviews. Anthropometric data were collected via physical measurements [28].

### 2.2. Dietary Assessment

A web-based 24 h dietary recall was administered at the end of the initial assessment visit for the last 70,000 recruited participants [29]. From February 2011 to April 2012, 320,000 participants who had provided an e-mail address were invited on four separate occasions to complete a 24 h dietary recall, the Oxford WebQ, of which 210,947 participants completed at least one. The Oxford WebQ covers 206 food items and 32 beverage items commonly consumed in the UK. Intakes were reported in standard units of measurement, e.g., servings, cups, slices, etc., with intake categories ranging from 0 to 3+ units [30]. The Oxford WebQ has been validated against interviewer-based 24 h dietary recalls showing acceptable correlations for total energy intake, and most nutrients and biomarkers showing acceptable correlations between the average values of two or more Oxford WebQs and the estimated true intakes of total energy, total sugar, potassium and protein [31,32].

A total of 79 food categories and 14 beverage categories from the Oxford WebQ has previously been defined using the UK National Diet and Nutrition Survey categories [30]. We used these food and beverage groups when defining the food groups used in the substitution analyses (Table S1). Legumes were defined as legumes and dietary pulses, baked beans, tofu-based products, peas, hummus, soy drinks and soy-based desserts and yogurt. Unprocessed red meat intake was defined as the intake of beef, pork, lamb or other meat, including offal. Processed red meat intake was defined as sausages, bacon (with and without fat), ham or liver paté. Total red meat was the combination of unprocessed and processed red meat. Other food groups included were animal-based foods, unhealthy plant-based foods, healthy plant-based foods and alcoholic beverages (Table S1). Animal-based and healthy and unhealthy plant-based food foods were grouped based on plant-based diet indices from previous studies [33,34,35,36].

As a single 24 h dietary recall does not assess habitual dietary intake and variation in diet over time at an individual level [37,38], only participants who completed two or more Oxford WebQs were eligible for inclusion in this study. Baseline food intakes were defined as average intakes from the reported 24 h diet recalls.

### 2.3. Liver Cancer Assessment

Liver cancer was defined according to ICD-10 diagnosis codes C22.0 for hepatocellular carcinoma (HCC) or C22.1 for intrahepatic cholangiocarcinoma (ICC) and ICD-9 diagnosis codes 1550, malignant neoplasm of liver, primary, or 1551, malignant neoplasm of intrahepatic bile ducts. Incident and prevalent cases of liver cancer and corresponding diagnosis dates were obtained via external linkage to central cancer registries or hospital inpatient episodes [39,40].

### 2.4. Assessment of Confounders

Confounders were defined a priori from a review of the literature and illustrated using directed acyclic graphs (Figure S1). The following confounding variables were selected: age, sex, educational level, Townsend deprivation index (TDI), living alone, physical activity, smoking, alcohol intake and waist circumference. Information on all confounders except age was collected at the initial assessment visit before the start of follow-up.

### 2.5. Statistical Analysis

Baseline characteristics and intake of food groups of all included participants and participants who developed liver cancer were described using standard summary statistics. Continuous variables were described with medians and interquartile ranges (IQRs, 25–75th percentiles) and categorical variables in total numbers (n) and percentage (%). Intakes of food groups were described in g/day.

Multivariable-adjusted Cox proportional hazards regression models were used to estimate hazard ratios (HRs) with corresponding 95% confidence intervals (CIs), with age as the underlying timescale. Participants were followed from the date of their last completed Oxford WebQ until the occurrence of the event of interest or due to right censoring, whichever came first. Participants were right censored in the event of death, loss to follow-up or administrative end of follow-up (31 October 2022).

The substitution analyses were conducted by modeling the replacement of an equal mass of meat with legumes. The portion size of the substitution was set to 15 g/day of legumes for 15 g/day of red meat to ensure that substitutions were below the mean intake of any of the substituted food groups in the cohort. The substitutions were modeled using the leave-one-out approach in which variables for every food group intake along with a variable for total food intake were included, except the food groups that were to be substituted [41]. To estimate replacing 15 g/day of all red meats (unprocessed and processed) with 15 g/day of legumes, the following model was defined:

(1) $$\begin{array}{cc} \log(h(t;x))= & \log\left(h_{0}\left(t\right)\right)+\beta_{1}\mathrm{Legumes}\;(15\;g/\mathrm{day})\;+\\ \; & \beta_{2}\mathrm{Total}\;\mathrm{food}\;\mathrm{intake}\;\left(g/\mathrm{day}\right)+\beta_{3}´\mathrm{Other}\;\mathrm{food}\;\mathrm{groups}\;\left(g/\mathrm{day}\right)+\beta_{4}´\mathrm{Covariates} \end{array}$$

When substituting only unprocessed red meat with legumes, processed red meat was added to the model:

(2) $$\begin{array}{cc} \log(h(t;x))= & \log\left(h_{0}\left(t\right)\right)+\beta_{1}\mathrm{Legumes}\;(15\;g/\mathrm{day})+\beta_{2}\mathrm{Processed}\;\mathrm{red}\;\mathrm{meat}\;(15\;g/\mathrm{day})\;+\\ \; & \beta_{3}\mathrm{Total}\;\mathrm{food}\;\mathrm{intake}\;\left(g/\mathrm{day}\right)+\beta_{4}´\mathrm{Other}\;\mathrm{food}\;\mathrm{groups}\;\left(g/\mathrm{day}\right)+\beta_{5}´\mathrm{Covariates} \end{array}$$

When substituting only processed red meat with legumes, unprocessed red meat was added to the model:

(3) $$\begin{array}{cc} \log(h(t;x))= & \log\left(h_{0}\left(t\right)\right)+\beta_{1}\mathrm{Legumes}\;(15\;g/\mathrm{day})+\beta_{2}\mathrm{Unprocessed}\;\mathrm{red}\;\mathrm{meat}\;(15\;g/\mathrm{day})\;+\\ \; & \beta_{3}\mathrm{Total}\;\mathrm{food}\;\mathrm{intake}\;\left(g/\mathrm{day}\right)+\beta_{4}´\mathrm{Other}\;\mathrm{food}\;\mathrm{groups}\;\left(g/\mathrm{day}\right)+\beta_{5}´\mathrm{Covariates} \end{array}$$

The performance of the leave-one-out model when modeling equal mass substitutions has been validated against simulated data [42].

Two levels of adjustments were added to the substitution model. Model 1 was minimally adjusted for age (as the underlying timescale), total weight of food and beverage intake (g/day) and all other food groups (g/day) to fit the substitution model. To account for differences in baseline risks, model 1 was additionally stratified on age at recruitment (<45, 45–49, 50–54, 55–59, 60–64 and ≥65), attended assessment center and sex (male, female). Model 2 was further adjusted for educational level (high: College or University degree; intermediate: A levels/AS levels, O levels/GCSEs, or equivalent; low: none of the previous mentioned), Townsend deprivation index (continuous), living alone (yes, no), physical activity (above/below the 2017 UK physical activity guidelines of 150 min of moderate activity per week or 75 min of vigorous activity, or unknown), smoking (pack years as a proportion of lifespan exposed to smoking, continuous), alcohol intake (g/day, continuous) and waist circumference (cm, continuous). The included covariates, including food groups, were grouped to ensure adequate power in the analyses to discover any associations between the exposure and outcome of interest and were guided by our a priori assumptions [26]. Assumptions of proportional hazards were checked using Schoenfeld residuals and showed no violations.

In secondary analyses, each cancer type was analyzed separately to evaluate if the pooling of HCC and ICC as one outcome in the main analysis was justified. Furthermore, to estimate the association of legume intake with liver cancer, not specifying the substitution, legume consumers (divided into quartiles) were compared to non-consumers.

To evaluate the robustness of the main analyses, sensitivity analyses were performed on subsamples of participants by excluding those with high alcohol intake (the exclusion of the upper decile of alcohol intake (g/day) by sex), implausible energy intake (the exclusion of participants below the 2.5th percentile and above the 97.5th percentile of energy intake (kJ/day) by sex), any liver disease before baseline, any type of cancer before baseline and fewer than three completed Oxford WebQs. As neither the central cancer registries nor the hospital inpatient registries were complete, liver cancer diagnoses retrieved from death registries, which were updated more recently, were included in a sensitivity analysis to test for outcome misclassification. Further, one of the causal assumptions was that anthropometry confounded the causal relationship between replacing red meat with legumes and liver cancer; however, arguments exist giving support to anthropometry being a mediator between diet and health outcomes. Thus, to test for erroneously conditioning on a potential mediator, a sensitivity analysis was adjusted following model 2 but without waist circumference. Lastly, sensitivity analyses omitting soy milk from the estimated daily legume intake were conducted, as soy milk is unlikely to culinarily replace red meat. All sensitivity analyses were modeled as the fully adjusted models in the main analyses.

All analyses were conducted in R version 4.4.0 (24 April 2024) with a significance level of 5%. The code is structured in a reproducible manner using the targets R package [43] and is available at https://github.com/steno-aarhus/legliv (accessed on 20 June 2024).

## 3. Results

**Figure 1:**
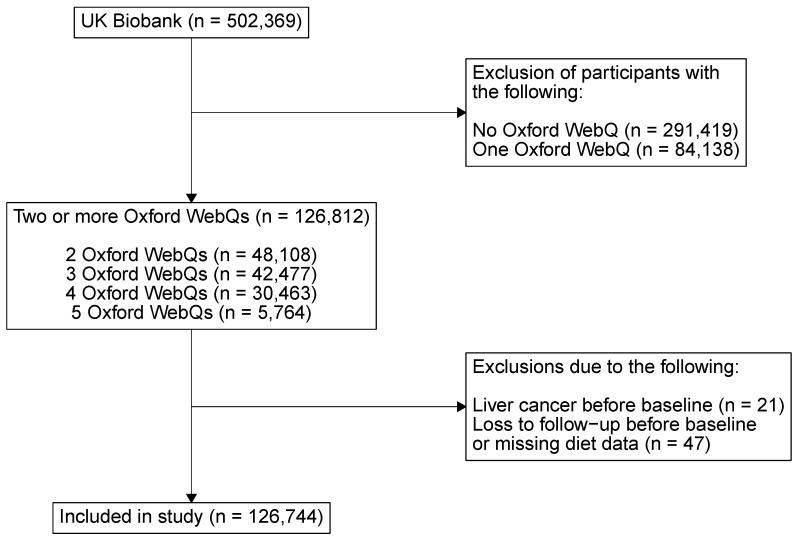
A flowchart of the included participants. Not all UK Biobank participants were invited to complete an Oxford WebQ. Only the last 70,000 participants to visit an assessment center were asked to complete an Oxford WebQ at the end of their visit. Further Oxford WebQs were sent to 320,000 participants who provided an e-mail address.

After excluding participants with liver cancer before baseline, participants lost to follow-up before baseline and participants with errors in dietary data, 126,744 participants who had completed two or more Oxford WebQs remained (Figure 1).

During a median follow-up time of 11.1 years, 173 participants developed liver cancer, of which 73 were HCC and 100 were ICC. Those who developed liver cancer were older at baseline, were more likely to be male, have a higher waist circumference and be less physically active, and fewer had never smoked, compared to all included participants (Table 1).

Mean daily energy and total food intakes as well as daily intake of all specified food groups in grams are presented in Table 2.

No evidence of associations was found between substituting 15 g/day of legumes with 15 g/day of total red meat, unprocessed red meat or processed red meat and the risk of primary liver cancer in model 1 (Table 3: total red meat: HR: 0.99, 95% CI: 0.93–1.05; unprocessed red meat: HR: 0.97, 95% CI: 0.91–1.03; processed red meat: HR: 1.04, 95% CI: 0.94–1.15). The estimated associations changed minimally with further adjustments. There was weak evidence of an association between the replacement of processed red meat with legumes (HR: 1.09, 95% CI: 0.99–1.21; Table 3).

In secondary analyses, when analyzing the associations between the replacement of red meat with legumes and HCC or ICC separately, weak evidence of a higher risk of HCC was observed (Table S2, total red meat: HR: 1.06, 95% CI: 0.97–1.16; unprocessed red meat: HR: 1.04, 95% CI: 0.95–1.15; processed red meat: HR: 1.10, 95% CI: 0.96–1.27). This association was opposite and inverse between the replacement of total red meat and unprocessed red meat and ICC (total red meat: HR: 0.97, 95% CI: 0.89–1.05; unprocessed red meat: HR: 0.94, 95% CI: 0.87–1.02) but not for processed red meat (HR: 1.07, 95% CI: 0.93–1.23, Table S2). The magnitude or direction of associations were not significantly different across strata of liver cancer types. In the adjusted non-substitution analysis, only the first quartile of legume intake (mean intake 6.3 g/day) was associated with a lower risk of liver cancer, compared to no intake (HR: 0.60, 95% CI: 0.36–0.99); no associations were observed for quartiles 2, 3 or 4 compared to no intake (Table S3). In sensitivity analyses, excluding participants based on high alcohol intake, implausible energy intake, any liver disease or cancer before baseline or fewer than three completed Oxford WebQs did not alter the estimates appreciably. Similar results were also found when including death registries as a source of liver cancer cases and when excluding waist circumference from the fully adjusted analysis and soy milk from the food substitutions (Table S4).

## 4. Discussion

Contrary to our hypothesis, this study showed little evidence of an association between replacing 15 g/day of unprocessed or processed red meat with legumes on risk of primary liver cancer. The estimates were robust to sensitivity analyses. When investigating liver cancer types separately, replacing total red meat and unprocessed red meat with legumes showed some weak evidence of an inverse association with ICC but with wide confidence intervals. The results for legume intake without specified substitutions did not show a clear pattern of associations.

The prospective longitudinal design of this study established temporality between diet exposure and liver cancer outcome, and the large sample size enabled analyses of a rare cancer. Further, our specified substitution analyses have some strengths in contrast to traditional methods in nutritional epidemiology through examining the association of consuming a food or nutrient while holding all other foods constant. The substitution is easily interpretable and reflects the implications that a higher intake of a food is at the expense of a lower intake of another food. A limitation of this research design was that the low intake of the substituted foods in this population restricted the size of the substitution, which may in turn have restricted findings of clinical relevance.

Information on dietary intake was collected using self-reported 24 h diet recalls, which may have introduced measurement error partly due to the limited ability of 24 h recalls to estimate habitual dietary intake. The lack of subsequently administered Oxford WebQs made it impossible to adjust for dietary changes during the study time. However, estimates were robust to the exclusion of participants with fewer than three completed Oxford WebQs, indicating that increasing the number of dietary measurements to account for some of the natural fluctuations in dietary intake over time made little difference to our results. A validation study of the Oxford WebQ found person-specific biases for correlation with true intakes for some nutrients, particularly for participants with a higher BMI [32]. Adjustment for BMI was not included in the current study. However, adjusting for waist circumference did not change the estimates appreciably, indicating that such errors do not explain our results. Finally, by specifying that the dietary exposure was collected on at least two occasions, the study population suffered considerable attrition. This is unlikely to be completely at random and most likely resulted in a study population with greater focus on their dietary habits compared to the general population. For example, the mean intake of processed meat was low in our study population. If a diet consisting of higher intakes of healthier plant-based foods is associated with lower liver cancer incidence, our study population may be at lower risk overall, thus reducing the power of our study to detect an association.

Registries used to determine a diagnosis of liver cancer were incomplete or not up-to-date at the time of analysis [39]. Data from external providers, such as the NHS England, NHS Central Register or National Records of Scotland, were estimated to be mostly complete by the UK Biobank at various dates, ranging from 31 December 2016 for cancer data from Wales to 31 October 2022 for hospital inpatient data from England [40]. This could introduce misclassification of the outcome, as individuals with liver cancer may not be identified as cases. However, the estimates were robust in a sensitivity analysis that included death registries as an additional source of liver cancer diagnoses to accommodate missing outcome events. Incorrectly classifying non-cases as cases would lead to attenuation of our results, but this is unlikely due to register linkage. Though health registries may have been only partially up-to-date, using registries almost eliminates selection bias due to loss to follow-up.

The relatively low number of events limited the possibility to adjust for confounding factors. Excessive adjustment parameters per event can compromise the validity of the multivariable Cox regression model, potentially causing biased estimates. To ensure statistical validity, at least 10 events per variable were aimed for in the main analysis by limiting the number of adjustment levels, using fewer and broader food groups and fewer levels for categorical covariates. This approach was guided by our a priori causal assumptions. Although this method helped maintain statistical validity, it may have increased residual confounding by diluting the importance of specific food groups. Additionally, risk factors that could not be adjusted for, such as aflatoxin B1, a known liver carcinogen, may have contributed to additional residual confounding.

Contrary to our hypothesis, replacing processed red meat with legumes was associated with a non-significant increase in the risk of primary liver cancer, with a greater effect size compared to unprocessed red meat. This pattern persisted across all sensitivity analyses. However, the estimates for processed red meat were labeled with less confidence, partly due to the low median intake. The findings of this current study align with other research in the UK Biobank, where unprocessed red meat intake was associated with a non-significant increase in liver cancer risk, with a greater effect size than processed meat (both white and red meat) [44]. This supports the notion that processed meat may not be associated with liver cancer risk in this population.

The literature on food substitutions, particularly in relation to liver cancer, is sparse. A recent meta-analysis of observational studies including approximately 350,000 individuals and 2125 liver cancer cases found a non-linear dose–response relationship between legume intake and liver cancer risk, with a protective effect observed between intakes of 8 g/day and 40 g/day [45]. This somewhat contrasts with our findings, where any increase above 6.3 g/day of legumes was not associated with a lower risk of liver cancer, compared to no legume intake. One recent meta-analysis of observational studies showed no association between red or processed meat intake and HCC [46], while another found a positive association between processed meat and HCC [47]. Another study examined the replacement of animal-based protein sources with plant-based protein sources and NAFLD risk in two cohorts and found a near-significant decrease in NAFLD when replacing processed meat, but not unprocessed red meat, with legumes in one cohort and a near-significant increase in NAFLD risk when replacing total red and processed meat with legumes in another cohort [25].

Red meat is the main source of exogenous heme iron, which catalyzes lipid peroxidation of LDL-cholesterol, leading to DNA damage [48]. Heterocyclic amines (HCAs) are formed when red meat is cooked at high temperatures and for a long time. Further, additives such as nitrate, nitrite and other N-nitroso compounds (NOCs) are often added in the processing of red meat and may, along with HCAs, constitute the carcinogeniticy of processed red meat [49,50,51]. On the contrary, legumes are high in dietary fibers which are linked to reduced risk of cardiovascular diseases and several cancers [52,53]. Despite the fact that the replacement of red meat with legumes will, inevitably, increase intake of dietary fibers and lower intake of possible carcinogens, this study found no association between the risk of liver cancer and this food substitution. Soy milk is low in fibers and did constitute a substantial amount of the legumes food group which would attenuate the difference in fiber intake and a possible beneficial association from replacing red meat with legumes. However, removing soy milk from food substitutions did not alter the results appreciably.

## 5. Conclusions

Overall, little evidence of an association between replacing red meat with legumes and liver cancer was observed. These results should be interpreted with caution due to the low intake of the substituted foods and few liver cancer cases. Further research in larger study populations with longer follow-up time is warranted. 

## Figures and Tables

**Table 1:** Baseline characteristics of UK Biobank participants who completed ≥2 Oxford WebQ dietary recalls.

	Cohort	Liver Cancer
Characteristic ^*1*^	N = 126,744	N = 173
**Age, years**	60 (53, 65)	64.0 (60.0, 68.0)
**Sex**		
Female	70,659 (56%)	65 (38%)
Male	56,085 (44%)	108 (62%)
**Educational level ^***2***^**		
High	59,416 (47%)	76 (44%)
Intermediate	41,817 (33%)	52 (30%)
Low	25,472 (20%)	45 (26%)
Missing	39	
**Townsend deprivation index**	−2.4 (−3.8, 0.0)	−2.6 (−3.7, −0.7)
Missing	149	
**Living alone**	22,658 (18%)	34 (20%)
Missing	171	
**Physical activity ^***3***^**		
Above	58,111 (46%)	61 (35%)
Below	50,712 (40%)	79 (46%)
Unknown	17,921 (14%)	33 (19%)
**Smoking**		
Never	72,583 (57%)	75 (43%)
Ever	54,122 (43%)	98 (57%)
Missing	39	
**Alcohol intake, g/day**	11 (0, 26)	11 (0, 29)
**Waist circumference, cm**	88 (79, 97)	98 (89, 107)
Missing	168	

^*1*^ Median (Q1, Q3) for continuous variables; n (%) for categorical variables. Variables are in bold. ^*2*^ High: College or University degree; intermediate: A levels/AS levels, O levels/GCSEs, or equivalent; low: none of the previously mentioned. ^*3*^ Above or below the 2017 UK physical activity guidelines of 150 min of moderate activity per week or 75 min of vigorous activity.

**Table 2:** Daily dietary intake of food groups, total food and total energy intake in UK Biobank participants who completed ≥ 2 Oxford WebQ dietary recalls.

	Cohort	Liver Cancer
Daily Food Intake ^*1*^	N = 126,744	N = 173
**Total food intake**		
Energy, kJ	8430 (7179, 9856)	8579 (7413, 10,048)
Weight, g	3144 (2720, 3621)	3162 (2737, 3659)
**Food groups, g/day**		
Legumes	11 (0, 34)	8 (0, 35)
Red and processed meat	53 (15, 86)	60 (30, 95)
Red meat	30 (0, 60)	45 (0, 73)
Processed meat	9 (0, 30)	8 (0, 31)
Other animal-based foods ^*2*^	475 (361, 603)	448 (322, 604)
Healthy plant-based foods ^*3*^	1806 (1454, 2198)	1791 (1365, 2158)
Unhealthy plant-based foods ^*4*^	472 (324, 662)	491 (365, 698)
Alcoholic beverages	132 (0, 342)	144 (0, 375)

^*1*^ Median (Q1, Q3). Variables are in bold. ^*2*^ Other animal-based foods include poultry, fish, dairy, eggs and mixed dishes with animal products. ^*3*^ Healthy plant-based foods include whole grains, vegetables, fruits, nuts, plant oils and beverages (coffee, tea, water). ^*4*^ Unhealthy plant-based foods include refined grains, potatoes, mixed vegetarian dishes, sweets and snacks, fruit juice and sugar-sweetened beverages.

**Table 3:** Replacing 15 g/day of total red meat, unprocessed red meat and processed meat with legumes and hazard ratios and 95% confidence intervals for primary liver cancer.

	Model 1 ^*1*^	Model 2 ^*2*^
15 g/day of Legumes Replacing:	HR (95% CI)	HR (95% CI)
Total red meat	0.99 (0.93–1.05)	1.02 (0.96–1.08)
Unprocessed red meat	0.97 (0.91–1.03)	1.00 (0.94–1.06)
Processed red meat	1.04 (0.94–1.15)	1.09 (0.99–1.21)

^*1*^ Multivariate Cox proportional hazards regression model adjusted for age (as underlying timescale), other food groups and total food intake, additionally stratified on sex, age and attended assessment center. ^*2*^ Further adjusted for educational level, Townsend deprivation index, living alone, physical activity, smoking, alcohol intake and waist circumference.

## Data Availability

This research has been conducted using the UK Biobank Resource under Application Number 81520. Data can be accessed via application to the Access Management System (AMS) at https://www.ukbiobank.ac.uk/enable-your-research/apply-for-access (accessed on 20 June 2024). A showcase of the data is available at https://biobank.ndph.ox.ac.uk/showcase/ (accessed on 20 June 2024).

## Supplementary Materials

The following supporting information can be downloaded at: https://www.mdpi.com/article/10.3390/nu16152383/s1, Figure S1: Simplified directed acyclic graph (DAG) visualizing the hypothesised causal relationship between replacing red meat with legumes and liver cancer based on assumptions of biasing paths; Table S1: Summary of included foods for each food group; Table S2: Replacing 15 g/day of total meat, red meat and processed meat with legumes and hazard ratios and 95% confidence intervals for hepatocellular carcinoma and intrahepatic cholangiocarcinoma; Table S3: No intake of legumes vs. quartiles of daily legume intake and hazard ratios and 95% confidence intervals for primary liver cancer; Table S4: Sensitivity analyses.

## Abbreviations

The following abbreviations are used in this manuscript:

DAG	Directed acyclic graphs
HCAs	Heterocyclic amines
HCC	Hepatocellular carcinoma
ICC	Intrahepatic cholangiocarcinoma
IQR	Interquartile range
NAFLD	Non-alcoholic fatty liver disease
NOCs	N-Nitroso compounds
TDI	Townsend deprivation index
