# Supplementary material for: The Association between the Substitution of Red Meat with Legumes and the Risk of Primary Liver Cancer in the UK Biobank: A Cohort Study
Source: Nutrients. 2024 Jul 23;16(15):2383. doi: 10.3390/nu16152383 (PMC11314238; doi:10.3390/nu16152383)
Supplement: Supplementary file 1 [file nutrients-16-02383-s001.zip › nutrients-3091662-supplementary-materials.pdf]

The Association between the Substitution of Red Meat with Legumes and the Risk of Primary Liver Cancer in the UK Biobank: A Cohort Study

Niels Bock [1], Fie Langmann [1], Luke W. Johnston [1,2], Daniel B. Ibsen [1,2], and Christina C. Dahm [1]

[1] Department of Public Health, Aarhus University, Aarhus, Denmark
[2] Steno Diabetes Center Aarhus, Aarhus University Hospital, Aarhus N, Denmark

June 20, 2024

Supplementary materials

Table S1. Summary of included foods for each food group.

Food group	Includes
**Legumes**	Soy-based desserts, baked beans, pulses, soy drinks (including calcium fortified), tofu-based products, hummus, peas
**Red meat**	Beef, lamb, other meat including offal, pork
**Processed meat**	Sausages, bacon (with and without fat), ham, liver pate
**Animal-based foods**	Poultry, fish, dairy, eggs, mixed dishes, and sauces and condiments
**Healthy plant-based foods**	Whole grains, fruits, nuts, plant oils, beverages (water, tea and coffee), vegetables
**Unhealthy plant-based foods**	Refined cereals, potatoes, fruit juice, mixed dishes (vegetarian), sweets & snacks, and sugar sweetened beverages
**Alcoholic beverages**	Beer and cider, spirits and other alcoholic drinks, fortified wine, red and rose wine, white wine

Table S2. Replacing 15 g/day of total meat, red meat and processed meat with legumes and hazard ratios and 95% confidence intervals for hepatocellular carcinoma and intrahepatic cholangiocarcinoma.

	Model 1[1]	Model 2[2]
**15 g/day of legumes replacing:**	**HR (95% CI)**	**HR (95% CI)**
**Hepatocellular carcinoma (n = 87)**		
Total red meat	1.02 (0.94-1.11)	1.06 (0.97-1.16)
Unprocessed red meat	1.02 (0.93-1.11)	1.04 (0.95-1.15)
Processed red meat	1.04 (0.90-1.19)	1.10 (0.96-1.27)
**Intrahepatic cholangiocarcinoma (n = 100)**		
Total red meat	0.94 (0.87-1.02)	0.97 (0.89-1.05)
Unprocessed red meat	0.92 (0.85-1.00)	0.94 (0.87-1.02)
Processed red meat	1.03 (0.90-1.18)	1.07 (0.93-1.23)

[1] Multivariate Cox proportional hazards regression model adjusted for age (as underlying timescale), other food groups, and total food intake, and additionally stratified on sex, age, and attended assessment centre.
[2] Further adjusted for educational level, Townsend deprivation index, living alone, physical activity, smoking, alcohol intake, and waist circumference.

Table S3. No intake of legumes vs. quartiles of daily legume intake and hazard ratios and 95% confidence intervals for primary liver cancer.

		Model 1[1]	Model 2[2]
**Characteristic**	**Mean daily legume intake**	**HR (95% CI)**	**HR (95% CI)**
Categories:			
No intake	0.00	—	—
Q1	6.3	0.59 (0.35-0.98)	0.60 (0.36-0.99)
Q2	16	0.88 (0.57-1.35)	0.90 (0.58-1.38)
Q3	34	0.73 (0.46-1.17)	0.74 (0.47-1.19)
Q4	109	0.98 (0.64-1.52)	1.07 (0.69-1.66)

[1] Multivariate Cox proportional hazards regression model adjusted for age (as underlying timescale), other food groups, and total food intake, and additionally stratified on sex, age, and attended assessment centre.
[2] Further adjusted for educational level, Townsend deprivation index, living alone, physical activity, smoking, alcohol intake, and waist circumference.

Table S4. Sensitivity analyses

	Exclusion of participants with:						Exclusion of:	
	**High alcohol intake**[1]	**Implausible food intake**[2]	**Liver disease before baseline**[3]	**Any cancer before baseline**[4]	**Fewer than 3 Oxford WebQs**[5]	**Death register as source of liver cancer events**[6]	**Waist circumference from analysis**[7]	**Soy milk from food substitutions**[8]
**15 g/day of legumes replacing:**	**HR (95% CI)**	**HR (95% CI)**	**HR (95% CI)**	**HR (95% CI)**	**HR (95% CI)**	**HR (95% CI)**	**HR (95% CI)**	**HR (95% CI)**
Total red meat	1.00 (0.94-1.06)	1.01 (0.95-1.07)	1.04 (0.96-1.12)	0.99 (0.93-1.06)	1.02 (0.96-1.08)	1.00 (0.94-1.06)	1.03 (0.96-1.11)	1.03 (0.94-1.12)
Unprocessed red meat	0.98 (0.92-1.05)	0.99 (0.93-1.05)	1.02 (0.94-1.11)	0.97 (0.90-1.04)	1.00 (0.94-1.07)	0.98 (0.92-1.05)	1.00 (0.93-1.08)	1.01 (0.92-1.11)
Processed red meat	1.06 (0.95-1.18)	1.08 (0.98-1.20)	1.11 (0.97-1.27)	1.08 (0.96-1.20)	1.07 (0.98-1.18)	1.06 (0.96-1.17)	1.15 (1.01-1.30)	1.11 (0.98-1.25)

[1] Exclusion of the upper decile of alcohol intake (g/day) by sex. n cases = 150.
[2] Exclusion of participants below the 2.5th percentile and above the 97.5th percentile of energy intake (kJ/day) by sex. n cases = 164.
[3] ICD10 codes: K70-79, B16-19, Z94.4, I85, I86.4, and E83.0-1. ICD9 codes: 5710-5745, 0700-0709, V427 and 2750-2751. n cases = 151.
[4] ICD10 codes: C00-C97 and D00-D48. ICD9 codes: 1400-2399. n cases = 129.
[5] n cases = 109.
[6] n cases = 183.
[7] n cases = 173.
[8] Soy milk was removed from the legumes food group and moved to the food group healthy plant-based foods. n cases = 173.

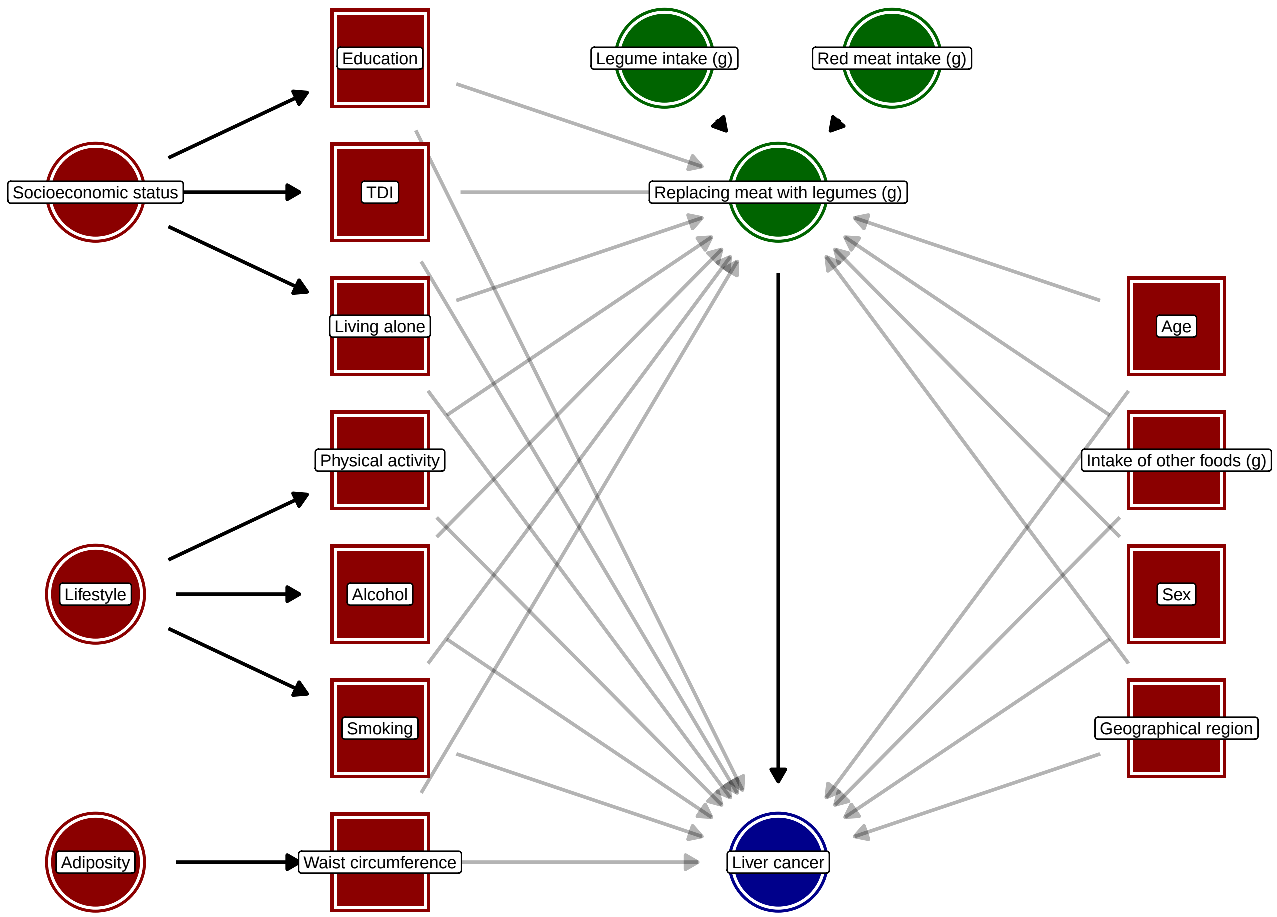

Figure S1. Simplified directed acyclic graph (DAG) visualizing the hypothesised causal relationship between replacing red meat with legumes and liver cancer based on assumptions of biasing paths. Red nodes represent confounders. Square nodes represent the minimal sufficient adjustment set for estimating the effect of replacing red meat with legumes on liver cancer. Shadowed arrows represent biasing paths. DAG terminology demands visualisation of all hypothesized correlating relationships between variables, typically resulting in complex and hard-to-follow illustrations. To improve readability, inter-covariate arrows are hidden in this DAG.
